# Analysis of the Damage and Failure Mechanism of Q345 Steel Plate with Initial Defect Under Different Temperature Conditions by Peridynamics

**DOI:** 10.3390/ma18081886

**Published:** 2025-04-21

**Authors:** Wudang Ying, Jinhai Zhao, Heipie Zhou, Yuchen Zhu, Yuquan Yang, Xinzan Hu

**Affiliations:** 1 Science and Technology Quality Department, China Construction Seventh Division (Shanghai) Co., Ltd., Shanghai 200031, China; 2 College of Mechanics and Engineering Science, Hohai University, Nanjing 211100, China; 3 Logistics and Infrastructure Department, Zhejiang Tongji Vocational College of Science and Technology, Hangzhou 311231, China; 4 Zhejiang Jiangnan Project Management Co., Ltd., Hangzhou 310007, China

**Keywords:** peridynamics, Q345 steel, bilateral cracks, bolt holes, damage and failure

## Abstract

The high temperature performance of steel structures has long been a focus of research, but research on the damage and crack propagation mechanism of steel with initial defects at high temperature is relatively low. The high temperature performance of most steel structures in engineering has an important impact on the function and safety of the whole structure. At present, Peridynamics (PD) theory uses the integral method that has unique advantages compared with traditional methods to solve structural damage and fracture problems. Therefore, the effect of temperature change on steel properties is introduced into the PD, and the PD constitutive equation at high temperature is proposed. The damage and crack propagation mechanisms of 2D Q345 steel plates with bilateral cracks and different bolt holes at 20 °C, 300 °C, 400 °C and 600 °C were analyzed by applying temperature action and external load to double-cracked steel specimens by the direct thermostructural coupling method. At the same time, the damage values, displacement changes in X direction and Y direction under different temperatures were compared and analyzed, and the effects of temperature, bolt hole and external load on the damage, displacement and crack growth path of different parts of the structure were obtained.

## 1. Introduction

The good ductility and shock resistance of steel make steel structure more and more popular in the field of construction engineering. However, the fire conditions lead to the reduction of the physical and mechanical properties of the steel, the reduction of the bearing capacity of the steel structure, and the acceleration of the destruction and collapse of the steel structure, such as when the temperature is 300 °C, the yield strength and elastic modulus of steel begin to decrease significantly. When the temperature is 400 °C, the yield strength is reduced to half of the room temperature, and the elastic modulus is reduced to about 60% of the room temperature. At 600 °C, the steel has significant plastic deformation [1]. For example, the light steel truss roof of the Prince George Airport in Canada collapsed in fire [2]; seventeen people have been killed in the fire at a terminal at Dusseldorf International Airport in Germany [3]; and a fire of department store in the Dezhou Shandong province China caused the steel roof collapsed [4]. Therefore, it is very important to clarify the high temperature bearing capacity and damage and failure mechanism of steel structures for fire resistance.

In the past two decades, the research methods and means of scholars from all over the world in the field of steel material properties and steel structure fire resistance have mainly manifested in work on the following areas: test analysis, single-member and whole structure calculation, and fire randomness [5]. The damage and failure mechanism of light steel structure under wind load is analyzed with a practical case, and various failure forms (joint failure, fatigue failure and instability of frame members) of steel structure joints are obtained [6]. AISI 4340 alloy specimens with a V-notch were tempered at different temperatures and analyzed by a four-point bending test. The specimens were characterized by J2 plastic theory and FEM model to determine the mechanism of influence of different factors on material damage and failure [7]. The mechanical properties of round, square and rectangular members under fire and after fire show that steel tube confined reinforced concrete columns have excellent fire resistance [8,9]. Through the connection between the results of substructure fire resistance test and finite element simulation analysis, the bearing limit of the structure under fire is given and the integrity of the structure is evaluated [10,11]. Based on the design goal of structural fire resistance, the research field of fire resistance of long-span spatial structures covers portal steel structure, grid structure, suspension cable structure and string beam structure. In addition, the mechanical property analysis method of long-span steel structure under fire has been proposed [12,13,14,15]. By collecting the high-temperature material parameters of Q345 steel, the sub-coefficient of resistance of Q345 steel axial bearing members at high temperature is calculated and presented with ABAQUS [16]. A study of Q345 cold-formed mechanical properties of steel under high temperature found that the tensile properties at peak temperature have the greatest influence on the material properties [17]. Another study established a finite element model of a three-dimensional elliptic hollow interfacial steel column under fire, and analyzed the effects of different constraints and slenderness ratio parameters on the stability of the structure [18]. The finite element model and machine learning framework have also been used to analyze the displacement corresponding to the steel structure roof under the condition of fire, and the calculation accuracy and efficiency are very consistent with the actual structure [19]. This has been combined with the finite element model and fluid mechanics to analyze the temperature change of steel structure under fire action and the corresponding structural dynamics [20]. A coupled non-local crystal plasticity and damage phase field model was established to analyze the strengthening and softening mechanisms of carbon steel structures under uniaxial tensile deformation, and the influence of internal structure and lattice structure on damage and failure was obtained [21].

In 2000, American scholars proposed the basic theory of PD using an integral algorithm [22]. Since then, PD theory has flourished, using uniform and non-uniform discretisation methods to determine the optimal horizontal dimensions based on ordinary and non-ordinary states under dynamic and static conditions [23]. The model of conservation of strain energy in deformation and fracture of solid materials is presented, and the non-local strain and crack nucleation damage characteristics of three types of cracks are analyzed [24]. In the field of steel or concrete, explored the damage of steel structures with porosity under impact load [25], proposed a new modeling method of concrete beam-column joint and concrete interface slip model, and analyzed the cracking problem of high performance concrete [26,27,28] and holes [29] on crack propagation are analyzed. At the same time, the failure characteristics of oil pipelines and concrete cubes under load have also been thoroughly studied [30,31]. Other research has established the finite element analysis model of concrete and steel structures under high temperatures, and has analyzed the damage and failure laws of structures under load [32,33]. The physical model and mechanical model of the bolt joint of steel structure have been established to analyze the damage and failure mechanism of the bolt and base metal under load [34].

Although the finite element method has great advantages in the establishment of complex models and the action of loads, it has many shortcomings in the analysis of structural damage and failure problems, such as the element fracture and crack propagation path, which depends on many parameters and the stress at the crack tip, which is infinite. PD theory perfectly solves the shortcomings of the finite element method and can accurately simulate the whole process of structural damage and destruction. It allows for analysis of the structural damage and failure characteristics of Q345 steel specimens with bilateral cracks and bolt holes at normal and high temperatures and better comprehends the damage and failure mechanism of steel structures at high temperatures. It also allows for analysis of temperature and bolt holes’ effect on displacement, damage, and crack growth in Q345 steel, providing theoretical support for high-temperature steel structure performance.

## 2. Basic Theory

The ISO 834 standard [35] temperature curve, $T_{g}=20+345\mathrm{lg}(8t+1)$, is used to calculate the variation of surface temperature of steel structure with time. Since the mechanical and physical properties of steel at high temperatures obtained by scholars from various countries are mostly semi-empirical formulas, the properties of high-temperature steel given by different scholars are very different, and the material parameters given by different scholars are described as follows.

### 2.1. Thermophysical and Thermodynamic Properties

(1) Thermal expansion coefficient $\alpha_{s}$

**Figure 1:**
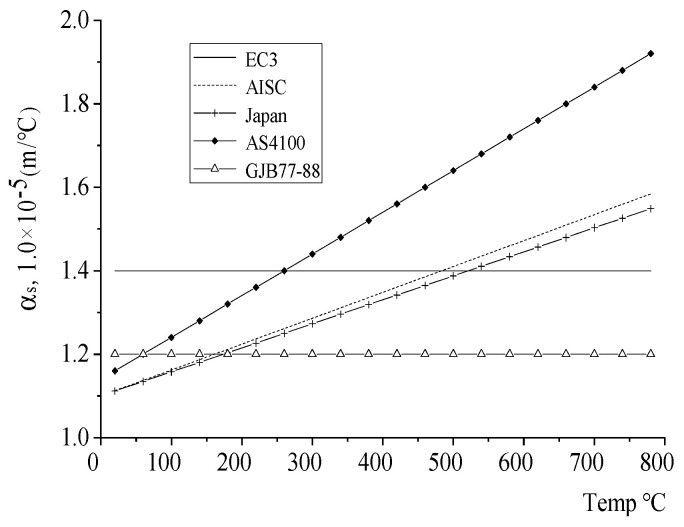
Thermal expansion $\alpha_{s}$.

The thermal expansion coefficient obtained by scholars from various countries through a large number of test data has a large difference in value, as shown in Figure 1. The results of the thermal expansion coefficient given by scholars from various countries are semi-empirical formulas obtained through experiments. The thermal expansion coefficient given by EC3 and GJB77-88 does not change with temperature and is a constant value. The coefficient of thermal expansion given by Japan, Australia, and other countries increases with the increase of temperature.

.

Through experiment and experience, Chinese scholars believe that the coefficient of thermal expansion of steel at high temperature is considered to be a reasonable fixed value, which can meet the actual situation and simplify the calculation process [32].

(1) $$\alpha_{s}=1.2\times 10^{-5}$$

(2) Elastic mode reduction coefficient

**Figure 2:**
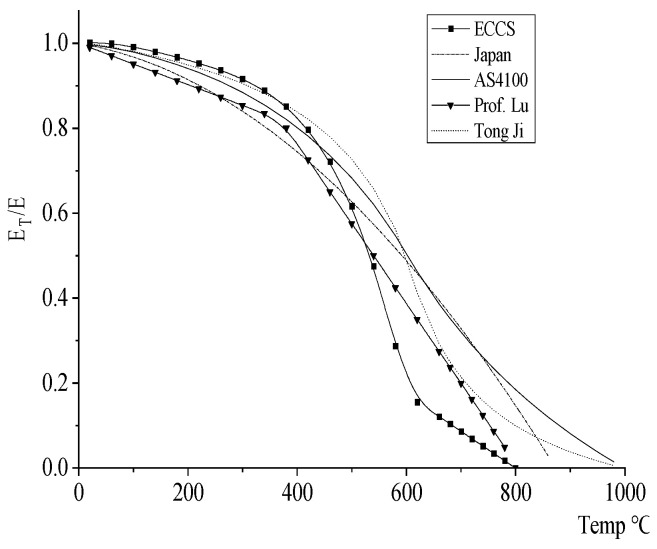
Elastic modulus reduction coefficient [36].

Before the temperature rises to 300 °C, the elastic modulus of steel is reduced by about 15%, and the change value is relatively small. When the temperature exceeds 300 °C, the elastic modulus of the steel begins to decrease significantly, and the cutting decline rate gradually accelerates. When the temperature rises to 500 °C, the elastic modulus decreases by about 50%, and the structure loses its bearing capacity when the temperature reaches 600 °C. The formulas for calculating the reduction coefficient of elastic modulus obtained by scholars from various countries through statistical analysis have certain differences, as shown in Figure 2. Among them, Tongji University gives the calculation method as follows [32].

(2) $$\frac{E_{T}}{E}=\frac{7T_{s}-4780}{6T_{s}-4760}\;\;\;20\;{}^{\circ}C\leq T_{s}\leq 600\;{}^{\circ}C$$

(3) $$\frac{E_{T}}{E}=\frac{1000-T_{s}}{6T_{s}-2860}\;\;\;600\;{}^{\circ}C\leq T_{s}\leq 1000\;{}^{\circ}C$$

(3) Density $\rho_{s}$

Scholars from all over the world agree that the density of steel $\rho_{s}$ is a constant value, and basically does not change with temperature [36].

(4) $$\rho_{s}=7850\;\mathrm{kg}/m^{3}$$

(4) Poisson’s ratio $\nu_{s}$

Scholars from all over the world have obtained that Poisson’s ratio of steel varies very little with temperature by experiments, and all of them agree that Poisson’s ratio is fixed value as temperature changing [36].

(5) $$\nu_{s}=0.3$$

### 2.2. Basic Theory of PD

**Figure 3:**
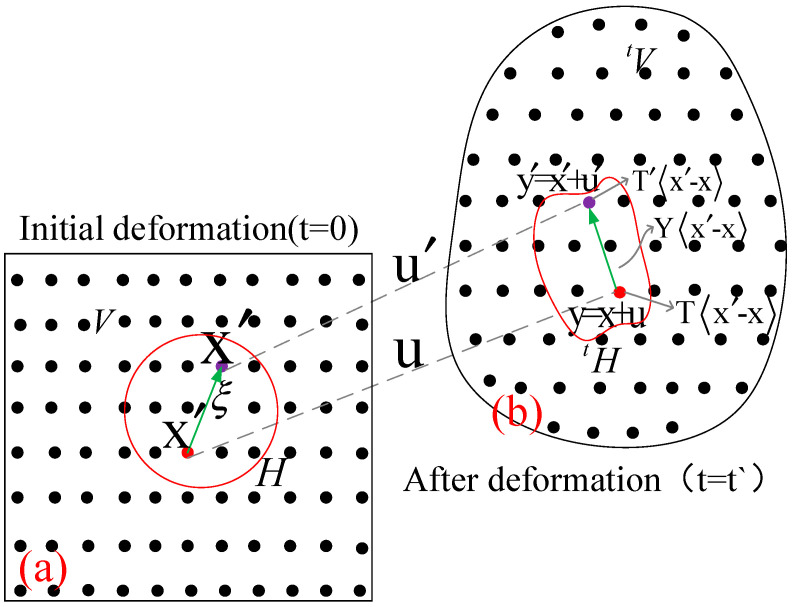
Interaction and deformation between adjacent particles. (**a**) Initial deformation; (**b**) After deformation.

The traditional methods mainly use tests and the finite element method to analyze the structural damage and crack propagation. However, these methods have some disadvantages in solving the problems of crack tip stress, structural meshing and stress field. The objects will discreet into particles x,y or k,j as per PD theory. This then determines the radius of interaction between particles and uses the integral algorithm to overcome the shortcomings of existing methods, as shown in Figure 3.

According to the interaction between particles, the scalar potential energy value $w_{\left(k)(j\right)}=w_{\left(k)(j\right)}\left(y_{\left(1^{k}\right)}-y_{\left(k\right)},y_{\left(2^{k}\right)}-y_{\left(k\right)},\cdots \right)$ and $w_{\left(j)(k\right)}=w_{\left(j)(k\right)}\left(y_{\left(1^{j}\right)}-y_{\left(j\right)},y_{\left(2^{j}\right)}-y_{\left(j\right)},\cdots \right)$ are proposed, and the expression of $w_{\left(k)(j\right)}\neq w_{\left(j)(k\right)}$ and potential energy is obtained by combining the interaction between particle $x_{\left(k\right)}$ and other particles in the range of action. The sum of strain energy density $W_{\left(k\right)}$ in the range of particle $x_{\left(k\right)}$ is obtained by integral.

(6) $$W_{\left(k\right)}=\frac{1}{2}\sum_{j=1}^{\infty }\left(\frac{1}{2}w_{\left(k)(j\right)}\right.\left.+\frac{1}{2}w_{\left(j)(k\right)}\right)\cdot V_{\left(j\right)}$$

As k=j, then $w_{\left(k)(j\right)}=0$.

In Equation (7). T is the sum of the kinetic energy of the object, expressed as $T=\sum_{i=1}^{\infty }\frac{1}{2}\rho_{\left(i\right)}{\dot{u}}_{\left(i\right)}\cdot {\dot{u}}_{\left(i\right)}V_{\left(i\right)}$; U is the sum of the potential energy of the object, written as $U=\sum_{i=1}^{\infty }W_{\left(i\right)}V_{\left(i\right)}-\sum_{i=1}^{\infty }\left(b_{\left(i\right)}\cdot u_{\left(i\right)}\right)V_{\left(i\right)}$. Through the principle of conservation of function and virtual work, the relationship between T and U at particle $x_{\left(k\right)}$ can be obtained.

(7) $$\delta \int_{t_{0}}^{t_{1}}\left(T-U\right)dt=0$$

The equilibrium relationship of PD particle $x_{\left(k\right)}$ force, physical force and external force is established, and the PD equilibrium equation is proposed.

(8) $$\rho_{\left(k\right)}{\ddot{u}}_{\left(k\right)}=\sum_{j=1}^{\infty }\left(\underline{T}(x_{\left(k\right)},t)-\underline{T}(x_{\left(j\right)},t)\right)V_{\left(j\right)}+b_{\left(k\right)}$$

**Figure 4:**
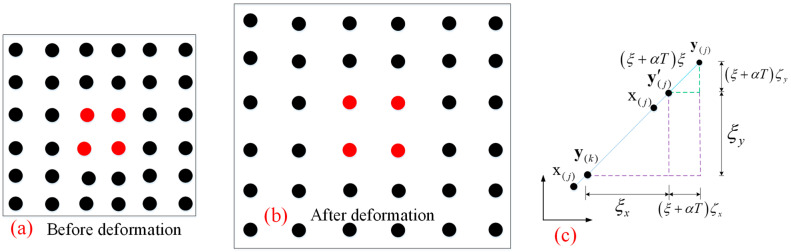
Isotropic expansion. (**a**) before deformation; (**b**) after deformation; (**c**) calculation model.

For two-dimensional isotropic materials subjected to tensile or expansion loads, as shown in Figure 4, the internal mechanical relations of stress and strain are obtained $\sigma_{\left(k\right)}^{T}=\left\{\sigma_{xx(k)}\;\sigma_{yy(k)}\;\sigma_{xy(k)}\right\}$, $\varepsilon_{\left(k\right)}^{T}=\left\{\varepsilon_{xx(k)}\;\varepsilon_{yy(k)}\;\varepsilon_{xy(k)}\right\}$. 

Then, the relationship between the material properties of two-dimensional objects can be expressed in the form of a matrix C.

(9) $$C=\left[\begin{array}{ccc} \frac{1}{\left(1-\nu^{2}\right)} & \frac{\nu }{\left(1-\nu^{2}\right)} & 0\\ \frac{\nu }{\left(1-\nu^{2}\right)} & \frac{1}{\left(1-\nu^{2}\right)} & 0\\ 0 & 0 & \frac{1}{2(1+\nu )} \end{array}\right]\cdot E$$

In which, κ represents bulk modulus, μ represents shear modulus, E represents for elastic modulus, and ν represents Poisson’s ratio, and $\kappa =\frac{E}{3(1-2\nu )},\;\mu =\frac{E}{2(1+\nu )}$.

For the two-dimensional classical continuum mechanics theory, the component of strain inside the body can be expressed as, $\varepsilon_{xx(k)}=\varepsilon_{\mathrm{yy}(k)}=\zeta +\alpha T$, and $\gamma_{xy(k)}=0$. The expansion term $\theta_{\left(k\right)}$ and the total strain energy $W_{\left(k\right)}$ inside the body can be expressed as $\theta_{\left(k\right)}=\varepsilon_{xx(k)}+\varepsilon_{yy(k)}=2\zeta +2\alpha T$, and $W_{\left(k\right)}=2\kappa \zeta^{2}$.

The relationship between particle $x_{\left(k\right)}$ and particle $x_{\left(j\right)}$ in a two-dimensional body with isotropic expansion by PD before and after deformation is $\left|y_{\left(j\right)}-y_{\left(k\right)}\right|=(1+\zeta +\alpha T)\left|x_{\left(j\right)}-x_{\left(k\right)}\right|$, then the expansion $\theta_{\left(k\right)}$ will be written as $\theta_{\left(k\right)}=\pi dh\delta^{3}\zeta +2\alpha T_{\left(k\right)}$, so the strain energy density $W_{\left(k\right)}$ are expressed as:

(10) $$\begin{array}{cc} W_{\left(k\right)} & =\;a\theta_{\left(k\right)}^{2}-a_{2}\theta_{\left(k\right)}T_{\left(k\right)}+a_{3}T_{\left(k\right)}^{2}\\ & +\;bh\int_{0}^{\delta }\int_{0}^{2\pi }\frac{\delta }{\xi }{\left(\left[\left(1+\zeta +\alpha T_{\left(k\right)}\right)\xi -\xi \right]-\alpha T_{\left(k\right)}\xi \right)}^{2}\xi d\theta d\xi \end{array}$$

By using classical continuum mechanics and PD theory, the expansion term $\theta_{\left(k\right)}$ and strain energy density $W_{\left(k\right)}$ of each expanding two-dimensional body are equivalent, and the expressions of PD theoretical parameters are obtained.

(11) $$4a+\frac{2}{3}\pi bh\delta^{4}=2\kappa$$

(12) $$a_{2}=4\alpha a,\;a_{3}=4\alpha^{2}a,\;d=\frac{2}{\pi h\delta^{3}}$$

**Figure 5:**
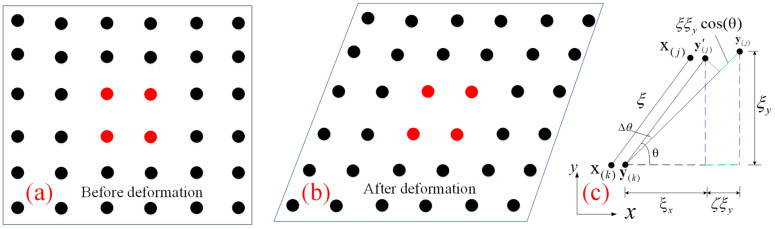
Shear problem. (**a**) before deformation; (**b**) after deformation; (**c**) calculation model.

The engineering shear strain $\gamma_{xy(k)}$ of classical continuum mechanics in two-dimensional pure shear objects is not zero, $\gamma_{xy(k)}=\zeta$, and the positive strain $\varepsilon_{xx(k)}=\varepsilon_{yy(k)}=\zeta =\alpha T=0$, as in Figure 5. So under pure shear condition, the expansion term $\theta_{\left(k\right)}$ of the object is zero, but the strain energy density term $W_{\left(k\right)}$ has value, $W_{\left(k\right)}=\frac{1}{2}\mu \zeta^{2}$.

The relationship between particle $x_{\left(k\right)}$ and particle $x_{\left(j\right)}$ in a two-dimensional body with isotropic expansion by PD after and before deformation is $\left|y_{\left(j\right)}-y_{\left(k\right)}\right|=\left[1+\left(\sin\theta \cos\theta \right)\zeta \right]\cdot \left|x_{\left(j\right)}-x_{\left(k\right)}\right|$, then the expansion $\theta_{\left(k\right)}$ will be written as $\theta_{\left(k\right)}=0$, the strain energy density $W_{\left(k\right)}$ calculated by integration can be expressed as $W_{\left(k\right)}=\frac{\pi h\delta^{4}\zeta^{2}}{12}b$.

Using the same analytical method as each expansion and comparing the shear strain energy and expansion direction obtained from classical continuum mechanics and PD theory, other parameters of the PD constitutive equation are deduced.

(13) $$a=\frac{1}{2}\left(\kappa -2\mu \right),\;b=\frac{6\mu }{\pi h\delta^{4}}$$

The classical continuum mechanics κ, μ and the material parameters of steel structure varying with temperature are introduced into the PD, and the parameters related to the bulk modulus and shear modulus are obtained.

(14) $$a_{T}=\frac{(3\nu -1)\cdot E_{T}}{4(1-\nu )(1+\nu )},\;a_{2T}=\frac{(3\nu -1)\cdot E_{T}\alpha }{\left(1-\nu )(1+\nu \right)},\;a_{3T}=\frac{(3\nu -1)\cdot E_{T}\alpha^{2}}{\left(1-\nu )(1+\nu \right)}$$

(15) $$b_{T}=\frac{3E_{T}}{\pi h\delta^{4}(1+\nu )},\;d_{T}=\frac{2}{\pi h\delta^{3}}$$

### 2.3. Elastic Crack Propagation Model

**Figure 6:**
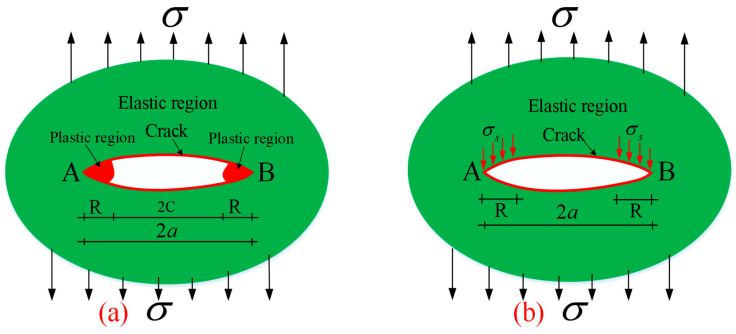
D-M analysis model of crack propagation. (**a**) Elastic-plastic model; (**b**) Elastic model.

In the process of crack tip opening, passivation, and expansion, there will be a local plastic region will appear at the crack tip, as shown in Figure 6a. Under the action of load, the red regions at the crack tips A and B inside the object are the plastic regions, while the rest of the material is an elastic region. The length of plastic region 2R at crack tips compared with crack length 2C, is that 2C >> 2R, and the material properties in the plastic region reach the limit state and can no longer bear the load. Therefore, the plastic region of the crack tip can be considered as a part of the crack, and the crack length changes from 2C to 2*a*, transforming the complex elastoplastic problem into an elastic problem, as shown in Figure 6b. Therefore, in this research, elastic theory is used to analyze the model and the crack.

## 3. Damage and Failure Mechanism of Q345 Steel Plates with Bilateral Crack and Bolt Holes Under Temperature

**Figure 7:**
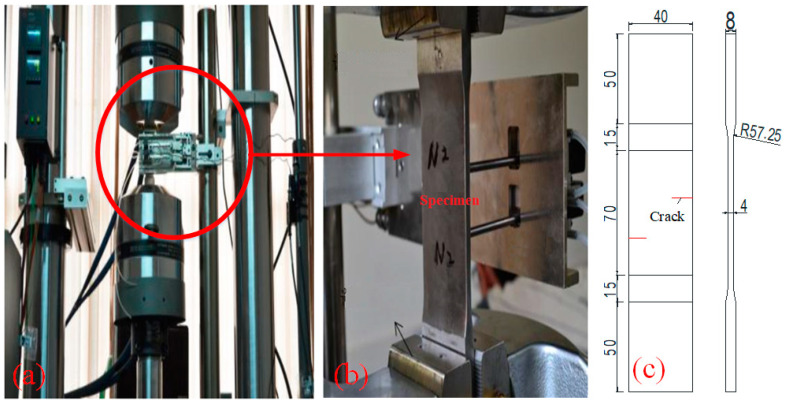
Test equipment and specimen model (unit: mm). (**a**) Test device; (**b**) Loading device; (**c**) specimen model.

Q345 was used in the test, and the main components were C (0.12~0.20%), Mn (1.00–1.60%), Si (0.20–0.60%), S ≤ 0.040%, P ≤ 0.040%. Elastic modulus E=203 GPa, Poisson ratio ν=0.3, and Density $\rho =7850\;{\mathrm{kg}/m}^{3}$ at room temperature. The experimental instrument is the electro-hydraulic servo tension testing machine MTS809 (XinGuang, Jinan, China). The experimental loading device, specimen loading process, and specimen model with initial crack are shown in Figure 7. Due to the limitations of test conditions and test equipment, only test analysis of specimens at normal temperature was carried out in this paper. Comparing normal temperature test results with theoretical data confirms that PD theory accurately simulates steel structure damage and failure, supporting its application to high-temperature cases. The experiments adopted the two-dimensional model, which means that PD theory can not only apply the thin plate model, but many three-dimensional models can be simplified into two-dimensional models, and two-dimensional models can also reflect the influence of different cracks or complex loads on the structure. The theoretical formulas given in Section 2.1 are used to calculate the physical and mechanical properties of steel under high temperature.

### 3.1. Analysis of the Influence of Bilateral Cracks on Crack Propagation Path

**Figure 8:**
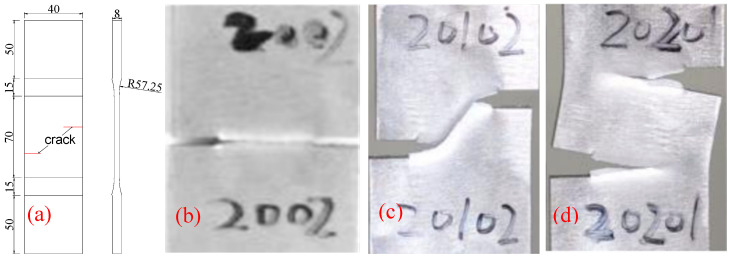
Specimen size and fracture result (mm). (**a**) Specimen size; (**b**) 2000×; (**c**) 2010; (**d**) 2020.

Bilateral crack tests with longitudinal spacing of 0 mm, 10 mm and 20 mm were made by Q345 steel. The crack is manufactured by wire cutting technology, and its width is 0.2 mm. The specimen numbers, crack longitudinal spacing, and size are all listed in Table 1. The crack location and longitudinal spacing are shown in Figure 8a. At room temperature 20 °C, a hydraulic testing machine was used to apply $2.217\times 10^{-5}\;m/s$ displacement load at both ends of the specimens, and the final fracture results of the three types of specimens are shown in Figure 8. It can be seen from the experimental fracture results of bilateral crack specimens that he longitudinal spacing of bilateral cracks plays a decisive role in the crack propagation path. The three types of specimens were, respectively, fractured into a 0 mm crack, a 45° oblique crack and two parallel cracks.

### 3.2. The Relationship Between Temperature and Crack Pacing on the Crack Paths

As can be seen from Figure 2, the elastic modulus of steel decreases gently before 300 °C, decreases by about half of the room temperature after 400 °C, and the steel basically loses its bearing capacity at 600 °C. Therefore, the effects of temperature on bilateral crack propagation paths and rates at different longitudinal spacing were analyzed at 20 °C, 300 °C, 400 °C and 600 °C.

**Figure 9:**
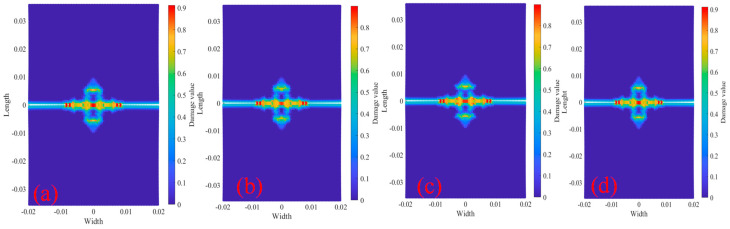
2000× tests crack propagation path at step 150. (**a**) 20 °C; (**b**) 200 °C; (**c**) 400 °C; (**d**) 600 °C.

Figure 9 shows the tests with longitudinal spacing of 0 mm and 2000× under displacement $2.217\times 10^{-5}\;m/s$ load at different temperatures. The two cracks finally fuse into a horizontal crack, but when the crack tip oscillates during the fusion process, the loading rate has a great influence on the crack tip oscillation, and the two cracks finally fuse into a horizontal crack. However, when the crack tip oscillates during the fusion process, the loading rate has a great influence on the crack tip oscillation, and the temperature has little effect on the crack fusion oscillation and the crack growth rate.

**Figure 10:**
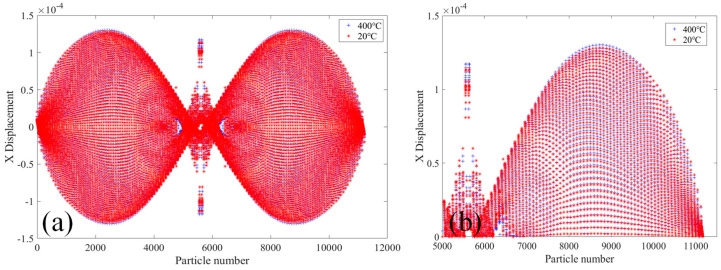
At 20 °C and 400 °C, the 2000× specimen’s displacement in the X-direction. (**a**) The whole specimen and (**b**) the top half of the specimen.

Figure 10 shows the comparative analysis of displacement changes in the X direction of the 2000× specimen at the temperatures of 20 °C and 400 °C. Figure 10a shows the X-direction displacement of PD particles of the whole specimen. It can be seen that the X-direction displacement of PD particles on both sides of the upper and lower ends of the specimen is opposite, and the X-direction displacement increases gradually along the middle of the two sections of the specimen, and the largest X-direction displacement at the position of 1/4 of the length of the specimen. In the middle part of the specimen, the PD particle displacement in the X-direction is the smallest, and the PD particle displacement in X-direction at the crack location suddenly increases. Figure 10b shows the X-direction displacement of PD particles in the upper part of the specimen. It can be seen that the X-direction displacement of PD particles at the specimen at 400 °C is slightly larger than that at 20 °C.

**Figure 11:**
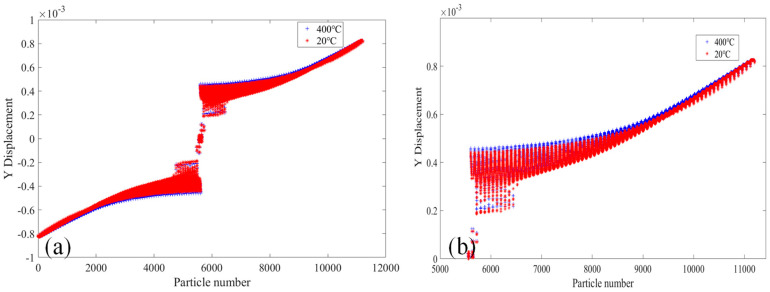
At 20 °C and 400 °C, the 2000× specimen’s displacement in the Y-direction. (**a**) The whole specimen and (**b**) the top half of the specimen.

Figure 11 shows the comparative analysis of the displacement change in the Y-direction of the 2000× specimen at the temperatures of 20 °C and 400 °C. Figure 11a shows the displacement in the Y-direction of the whole specimen’s PD particles. It can be seen that the displacement in the Y-direction of the PD particles of the lower part of the specimen moves downward, while the displacement in the Y-direction of the PD particles of the upper part of the specimen moves upward. The displacement in the Y-direction of the PD particles of the two sections of the specimen reaches a maximum and gradually decreases toward the center of the specimen. The displacement value of PD particles in the Y-direction changes abruptly in the crack expansion region of the specimen. Figure 11b shows the displacement of PD particles in the Y-direction of the upper part of the specimen. The displacement of PD particles in the Y-direction of the specimen at 400 °C is slightly larger than that at 20 °C.

**Figure 12:**
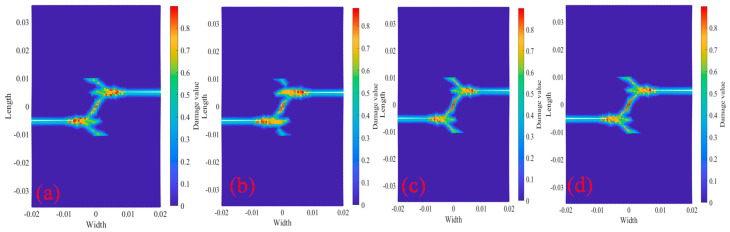
2010× tests eventually formed a 45° oblique crack at step 150. (**a**) 20 °C; (**b**) 200 °C; (**c**) 400 °C; (**d**) 600 °C.

As can be seen from Figure 12, under different temperatures, the double cracks are affected by the longitudinal spacing of 10 mm, and the two cracks first expand horizontally and then diagonally expand along 45° and fuse. The high temperature has a great influence on the damage degree of the specimen, but little influence on the crack propagation path.

**Figure 13:**
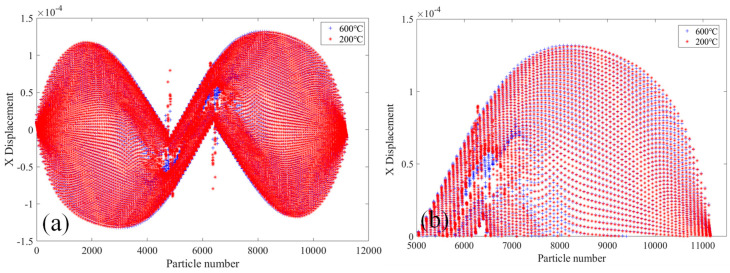
At 200 °C and 600 °C, the 2010× specimen’s displacement in X-direction. (**a**) The whole specimen; (**b**) the top half of the specimen.

Figure 13 shows the comparative analysis of the displacement change in the X-direction of the 2010× specimen at the temperatures of 20 °C and 400 °C. As shown in Figure 13a, both the X-direction displacement of the whole PD particles and the initial crack of the specimen are related to the skew symmetry of the specimen center. The X-direction displacement of PD particles in two sections of the specimen is the smallest and gradually increases toward the center of the specimen, and is the largest at about 1/4 of the length of the specimen. In the middle of the two initial cracks of the specimen, the PD particle displacements are close to 0 mm. Figure 13b shows the PD particle displacement in the X-direction of the lower half of the specimen. It can be seen that the PD particle displacement of the specimen in the X-direction at 600 °C is larger than the PD particle displacement at 20 °C.

**Figure 14:**
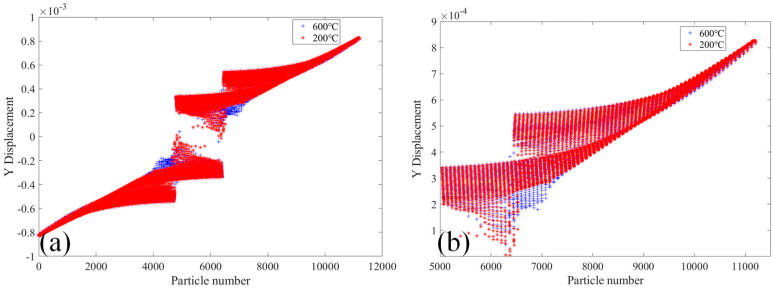
At 200 °C and 600 °C, the 2010× specimen’s displacement in the Y-direction. (**a**) The whole specimen; (**b**) the top half of the specimen.

Figure 14 shows the comparative analysis of the change in the Y-direction displacement of the 2010× specimen at 20 °C and 600 °C. It can be seen in Figure 14a that under the two conditions, the change trend of Y direction displacements of the specimen’s PD particles is constant, and the Y direction displacement value of PD particles oscillates and changes in a step form at the crack deflection position. Figure 14b shows the Y-direction displacements of PD particles in the upper part of the specimen. It can be found more obviously that the change of PD particles Y-direction displacement of the specimen under the action of two different temperatures.

**Figure 15:**
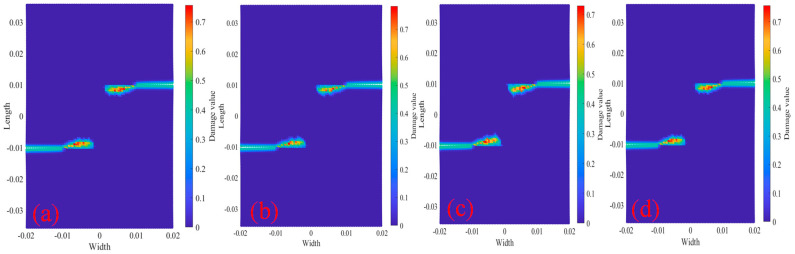
2020× tests formed two parallel cracks at step 150. (**a**) 20 °C; (**b**) 200 °C; (**c**) 400 °C; (**d**) 600 °C.

As shown in Figure 15, at different temperatures, cracks of 2020× tests with longitudinal spacing of 20 mm propagate into two parallel horizontal cracks under displacement $2.217\times 10^{-5}\;m/s$ load. It can be seen from Figure 15 that temperature does not affect the crack propagation path of the tests, while from the damage diagram that temperature has a great influence on the damage rate.

**Figure 16:**
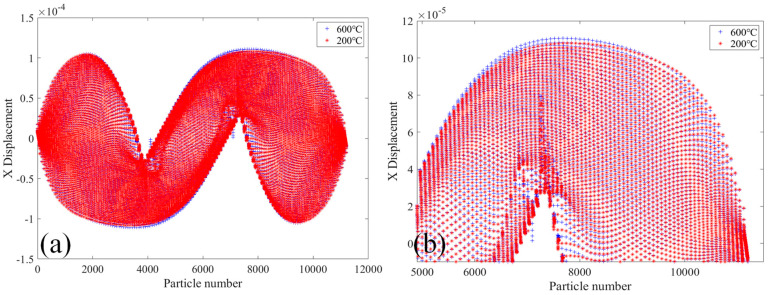
The figure shows the 2020× specimen’s displacement in the X-direction at 200 °C and 600 °C. (**a**) The whole specimen; (**b**) the top half of the specimen.

Figure 16 shows the comparative analysis of displacement changes in the X direction of the 2020× specimen at 20 °C and 400 °C. By comparing Figure 16 and Figure 13, it can be seen that the displacements of PD particles in the X-direction of the two types of specimens are similar. The PD particles of specimen 2010× are around the numbers 4300 and 6300, and the displacements in the X-direction are abrupt, while the PD particles of specimen 2020× are around the numbers 4000 and 7000. In both cases, PD particle displacements in the X-direction in the middle region of the two initial cracks of the specimen are close to 0 mm. Figure 16b shows the PD particle displacement in the X-direction of the upper part of the 2020× specimen. It can be seen that the PD particle displacement in the X direction of the specimen at 600 °C is larger than the PD particle displacements at 20 °C.

**Figure 17:**
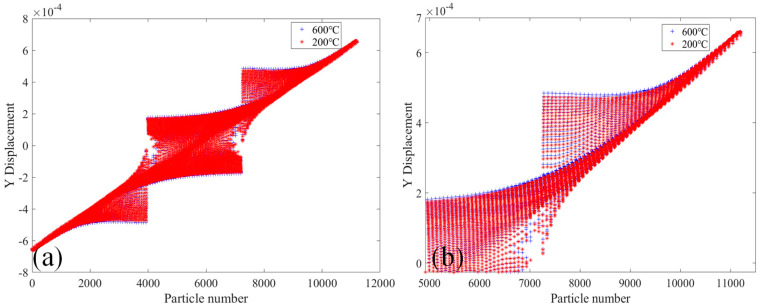
The figure shows the 2020× specimen’s displacement in the Y-direction at 200 °C and 600 °C. (**a**) The whole specimen; (**b**) the top half of the specimen.

Figure 17 shows the comparative analysis of displacement change in the Y-direction of the 2020× specimen at 20 °C and 600 °C. As can be seen in Figure 17a, the displacements in the Y direction of the PD particles of specimen 2020× are skew-symmetric with respect to the center of the specimen. The displacements in the Y-direction in two sections of the specimen are the largest and gradually decrease toward the center of the specimen. Figure 17b shows the displacement value of PD particles in the Y-direction of the upper half part of the specimen. The change in the displacement value of each PD particle in the Y direction can be inferred that the displacements of PD particles in the Y-direction of the specimen at 600 °C are greater than those at 200 °C.

**Figure 18:**
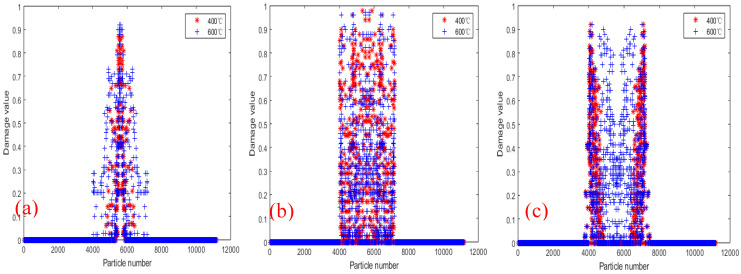
Damage comparison of tests at temperatures of 400 °C and 600 °C. (**a**) Test 2000×; (**b**) Test 2010×; (**c**) Test 2020×.

Figure 18 shows the PD particle damage rate results of tests 2000×, 2010× and 2020× under displacement $2.217\times 10^{-5}\;m/s$ load at 400 °C and 600 °C. It can be seen that the damage rate and damage range of the three tests at 600 °C are higher than those at 400 °C. The results show that although temperature has no significant effect on the crack propagation path of bilateral crack tests, the increase in temperature decreases the physical and mechanical properties of materials, and accelerates the crack propagation rate and local expansion range.

### 3.3. The Relationship Between Temperature and Bolt Holes on the Crack Paths

At present, the steel structure is mostly connected by bolts, and some bolt holes need to be made on the steel plate. When a fire or high temperature occurs in the working environment of the steel structure, the crack propagation path of the Q345 bilateral crack steel plates will change greatly under the influence of high temperature and bolt holes. Understanding the impact of high temperature and bolt holes on the damage and failure of the steel structure is crucial to the high-temperature bearing capacity of the steel structure. Different types of Q345 bolt joint specimens can be made, as shown in Table 2.

**Figure 19:**
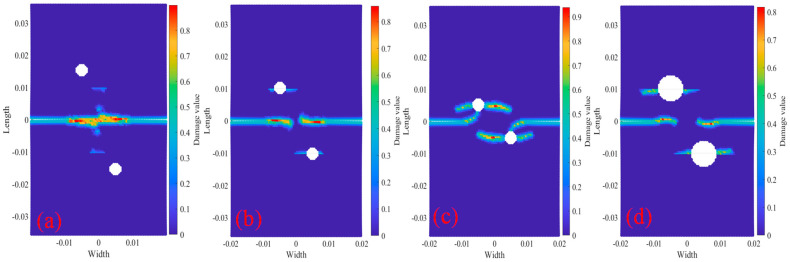
Analysis the damage and crack propagation of tests 2000× with bolt holes at 20 °C. (**a**) 2000R2D30; (**b**) 2000R2D20; (**c**) 2000R2D10; (**d**) 2000R4D20.

Figure 19 shows the bilateral crack with longitudinal spacing of 0 mm at 20 °C, including two tests with bolt holes, and the crack propagation path under load (where R represent the radius of bolt holes and D represent the longitudinal distance between two bolt holes). The center coordinate of the left bolt hole is O_1_ (x_1_ = −0.005 m), and the center coordinate of the right bolt hole is O_2_ (x_2_ = 0.005 m). By comparing Figure 9 with Figure 19a, it can be seen that (y_1_ = 0.0015 m, y_2_ = −0.015 m), when the bilateral crack tests with longitudinal spacing 0 mm is fused into a horizontal crack, it is affected by the screw hole, and the local damage range of the final fusion area of the two cracks is large. It can be seen from Figure 19b that the center of the circle coordinates O_1,2_ (y_1_ = 0.001 m, y_2_ = −0.01 m), horizontal damage occurs on both sides of the holes when the two cracks fuse. It can be seen from Figure 19c that the center of the circle coordinates O_1,2_ (y_1_ = 0.0005 m, y_2_ = −0.005 m), when the two horizontal crack tips extend below the bolt holes, the crack tips extend toward the center of the bolt holes. It can be seen from Figure 19d that, at the center of the circle coordinates O_1,2_ (y_1_ = 0.001 m, y_2_ = −0.01 m), the expansion path of the two horizontal cracks bends slightly during the propagation process, but basically maintains straight line expansion, and the horizontal damage trend on both sides of the bolt hole are serious. It can be seen from the hole damage and crack propagation of four kinds of bolts in Figure 19 that the hole location and radius have a great influence on structural damage.

**Figure 20:**
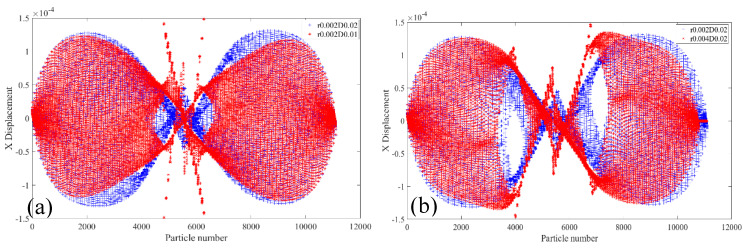
Displacements of PD particles in the X-direction at 20 °C. (**a**) 2000R2D10 and 2000R1D20; (**b**) 2000R2D20 and 2000R4D20.

Figure 20a shows the displacements of 2000R2D10 and 2000R1D20 specimens containing two bolt holes with a radius of 2 mm and longitudinal spacing of 10 mm and 20 mm in the X-direction at 20 °C. The displacements in the X direction of the center area of the specimen and the maximum displacements in the X direction of the specimen with the bolt hole longitudinal spacing of 10 mm are smaller than the displacement in the X direction of the corresponding area of the bolt hole of 20 mm. Figure 20b shows that two specimens 2000R2D20 and 2000R4D20 with radius of 2 mm and 4 mm, and a spacing of 20 mm, are displacements in the X-direction at 20 °C. The change law of the displacement in the X-direction of the two specimens is consistent with the change law of the PD particle in the X-direction of the two specimens in Figure 20a.

**Figure 21:**
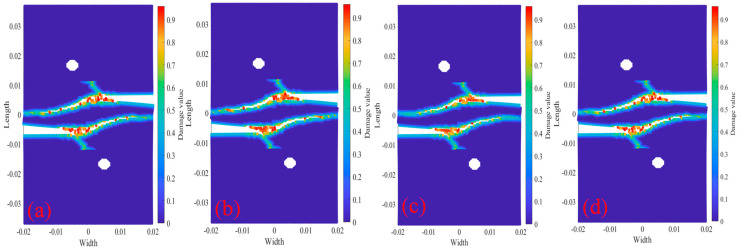
Damage and crack propagation paths of step 350 tests 2010R2D30. (**a**) 20 °C; (**b**) 200 °C; (**c**) 400 °C; (**d**) 600 °C.

Figure 21 shows the Q345 bilateral crack tests with longitudinal spacing of 10 mm, including two bolt holes with a radius of 0.002 m. The center coordinates of bolt holes is O_1_ (x_1_ = −0.005 m, y_1_ = 0.015 m) and the center coordinates of bolt holes is O_2_ (x_2_ = 0.005 m, y_2_ = −0.015 m). The figure also shows bolt hole damage and crack propagation path under displacement load $2.217\times 10^{-5}\;m/s$ and temperature. The figure shows that the crack propagation path is basically the same under the action of the four temperatures. It can be inferred that the multi-horizontal crack propagation path of temperature has little influence. As can be seen from the comparison of Figure 12 and Figure 21, the bilateral crack propagation path with a longitudinal distance of 10 mm between the bolt holes and the non-bolt holes changed greatly. The crack of the non-bolt holes tests the eventual propagation into a 45° oblique crack, and the bilateral cracks of the bolt holes test the eventual propagation into two curved cracks with a gradually smaller longitudinal distance.

**Figure 22:**
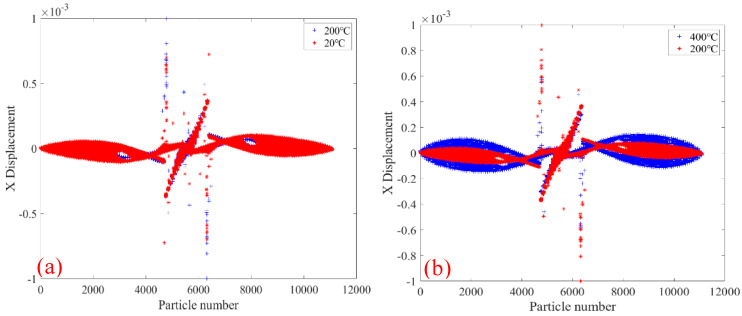
Displacements of 2010R2D30 specimen in the X-direction under different temperatures. (**a**) 20 °C and 200 °C; (**b**) 200 °C and 400 °C.

Figure 22 shows the displacements of PD particles in the X-direction of specimen 2010R2D30 under load with two bolt holes, radius R = 2 mm and longitudinal spacing D = 30 mm, and an initial crack with a length of 10 mm. Figure 21a shows the PD particle displacement in the X direction of 2010R2D30 specimen at 20 °C and 200 °C. It can be seen that the displacement trend and position value of PD particles in the X direction of the specimen at the two temperatures are basically the same, and the displacement value of PD particles in the X-direction changes abruptly in the crack region. Figure 22b shows the X-direction displacements of PD particles of the specimen at 200 °C and 400 °C. It can be seen that temperature has little effect on the X-direction displacements of 2010R2D30PD particle of the specimen at 400 °C.

**Figure 23:**
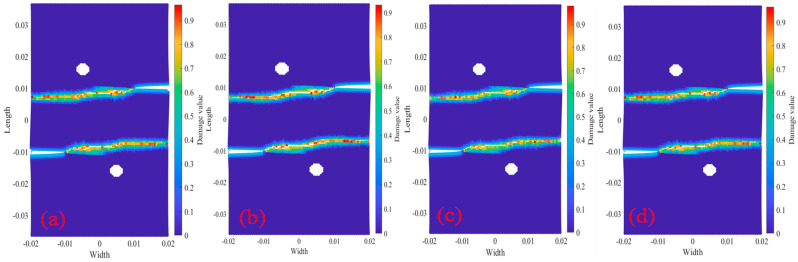
Damage and crack propagation paths of step 350 tests 2020R2D30. (**a**) 20 °C; (**b**) 200 °C; (**c**) 400 °C; (**d**) 600 °C.

Figure 23 shows the Q345 specimens 2020R2D30 with bilateral crack longitudinal spacing 20 mm, including two bolt holes with a radius of 0.002 m. One center coordinates of the bolt holes are O_1_ (x_1_ = −0.005 m, y_1_ = 0.015 m), and the other center coordinates of the bolt holes are O_2_ (x_2_ = 0.005 m, y_2_ = −0.015 m). According to the results of crack propagation at other tests, under the action of four temperatures, the crack propagation paths are basically the same, and the bilateral crack eventually expands into two parallel horizontal cracks.

**Figure 24:**
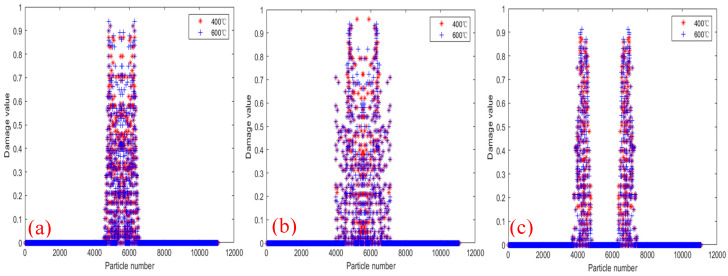
Damage comparison of tests with bolt holes at temperatures of 400 °C and 600 °C. (**a**) 2000R2D10; (**b**) 2010R2D = 30; (**c**) 2020R = 2D = 30.

Figure 24 shows the comparison of PD particle damage rates of specimens with bolt holes at 400 °C and 600 °C. Figure 24a shows the comparison results of the damage rates of test 2000R2D10. The damage locations of the tests are mainly concentrated in the center of the specimens. Figure 24b is the comparison result of the damage rate of tests 2010R2D30. It can be seen that the longitudinal distance of the crack decreases, and there is a bifurcation phenomenon during crack propagation. Figure 24c shows the comparison result of the damage rate of test 2020R2D30. It can be seen that the crack paths are not affected by the bolt holes, and propagation paths along the straight line, and finally form two parallel cracks. The comparative analysis of the damage rates of the three specimens shows that the PD particle damage rate of the 600 °C specimen is slightly higher than that of the 400 °C. The higher the temperature, the faster the particle damage rate and the larger the damage range.

## 4. Conclusions

This paper analyzes a single steel member. Future work will study high-temperature thermal expansion effects on structural deformation, fatigue, and linkage. However, the rule of material properties of steel structure varying with temperature is introduced into PD, and then PD parameters and constitutive equations varying with temperature are derived. The crack propagation path of bilateral crack tests was understood through experiments, and the damage and crack propagation mechanism of tests at 20 °C, 200 °C, 400 °C and 600 °C were analyzed under the joint action of temperature and bolt holes.

1. At room temperature 20 °C, the tests with bilateral crack longitudinal spacing of 0 mm, 10 mm, and 20 mm, showed crack propagation forming into a horizontal crack, a 45° oblique crack and two horizontal penetration cracks. It shows that the longitudinal crack spacing has a great influence on the crack propagation path.
2. The damage and crack propagation path of the same tests under different temperatures are basically the same, but the particle damage rate is different. It can be seen that the temperature has relatively little influence on the crack propagation path, but has a greater influence on the material damage rate, and the higher the temperature is, the faster the damage rate will be.
3. Under the same initial crack conditions and different bolt holes, the damage, PD particle displacement in X- or Y-direction and crack growth path of the specimen changed greatly. The results show that the location and radius of bolt hole have great influence on the damage, PD particle displacement and crack propagation path.
4. Through the results of temperature and bolt holes on the crack propagation rate and path of the tests, it can be concluded that a reasonable bolt hole radius and position are very important to the bearing capacity of steel structures.
5. As the temperature increases, the material properties of the steel are reduced, resulting in a decrease in the bearing capacity of the steel structure. Therefore, under the action of load, the higher the temperature, the larger the damage value of the specimen, and the damage parts will also be greatly different. At the same time, the displacement values in the X-direction and the Y-direction change faster under high temperature than under normal temperature. Therefore, steel structure buildings need to take fire protection measures to delay the heating rate of the structure under the action of temperature, and to improve the bearing capacity and the safety performance of the structure.

## Figures and Tables

**Table 1:** Specimen information.

Crack Length (mm)		Crack Spacing and Specimens No.		
10 (left)	10 (right)	0 (2000×)	10 (2010×)	20 (2020×)

**Table 2:** Specimens information (mm).

Crack Length and Longitudinal Spacing		Bolt Hole Radius(r) and Longitudinal Spacing(D)		Specimen No.
L and R Lengths	Spacing	Bolt Hole Radius	Spacing	
10, 10	0	R = 2	D = 10	2000R2D10
10, 10	0	R = 2	D = 20	2000R2D20
10, 10	0	R = 2	D = 30	2000R2D30
10, 10	0	R = 4	D = 20	2000R4D10
10, 10	10	R = 2	D = 30	2010R2D30
10, 10	20	R = 2	D = 30	2020R2D30

## Data Availability

The original contributions presented in this study are included in the article. “Further inquiries can be directed to the corresponding authors” or “The raw data supporting the conclusions of this article will be made available by the authors on request”.

## Nomenclature

The following symbols are used in this paper:

$a,a_{2},a_{3},b,d$	PD parameters at room temperature [Dimensionless quantity]
$a_{T},a_{2T},a_{3T},b_{T},d_{T}$	PD parameters at high temperature [Dimensionless quantity]
$\alpha_{s}$	Coefficient of thermal expansion of steel [Dimensionless quantity]
$b_{\left(k\right)}$	Body load vectors at particle k [N/m^3^]
C	Two-dimensional material property matrix [GPa]
$E,E_{T}$	Elastic modulus of steel at normal temperature and high temperature [GPa]
κ	Bulk modulus [GPa]
t	The time of the fire burns [s]
$T_{g}$	The temperature varies with the fire [°C]
$T_{s}$	Internal temperature of steel structure [°C]
T	The sum of the kinetic energy of the object, or temperature load [J, °C]
μ	Shear modulu [GPa]
${\dot{u}}_{\left(i\right)}$	The displacement vector of particle *i* [m/s]
${\ddot{u}}_{\left(k\right)}$	The acceleration of particle k [m/s^2^]
U	The sum of the potential energy of the object [J]
$\nu_{s}$	Poisson’s ratio of steel [Dimensionless quantity]
$V_{\left(j\right)}$	The volume of particle *j* [m^3^]
$w_{\left(k)(j\right)}$	Micro-potential energy depends on the state of material points within the family of material point $x_{k}$ and $w_{\left(k)(j\right)}\neq w_{\left(j)(k\right)}$ [J/m^3^]
$W_{\left(k\right)}$	The strain energy density of particle $x_{\left(k\right)}$ [J/m^3^]
$x_{\left(k\right)}$	Particle k or Displacement in x direction [m]
$y_{\left(k\right)}$	The deformed coordinate of particle $x_{\left(k\right)}$ [m]
δ	The radius of the particle force [m]
$\rho_{s}$	Density of steel [kg/m^3^]
ζ	Positive strain [Dimensionless quantity]
$\sigma_{\left(k\right)}$,$\varepsilon_{\left(k\right)}$	The stress and strain of the object [MPa]
$\theta_{\left(k\right)}$	Expansion term [Dimensionless quantity]
PD	Peridynamics
